# Supplementary material for: Identification of novel BRCA1 large genomic rearrangements by a computational algorithm of amplicon-based Next-Generation Sequencing data
Source: PeerJ. 2019 Nov 15;7:e7972. doi: 10.7717/peerj.7972 (PMC6859874; doi:10.7717/peerj.7972)
Supplement: Table S2 — F: forward strand; R: reverse strand. [file peerj-07-7972-s002.docx]

| **genomic variant** | **Primers sequence** * |
| --- | --- |
| NG_005905.2: g.163181_169408del6228 (exon 21-22 del) | F: TGCAAGATTTCACACTTACCTGT  R: ATGGAAGCCATTGTCCTCTG |
| NG_005905.2: g.160396_164568del4173 (exon 20 del) | F: GGTCTCAAGCTGTCCTCCTG  R: GTGCCACTCTCACTACCTGT |
| NG_005905.2:g.145185_151339del6155 (exon 16-17 del) | F: GGTTGTTGATGTGGAGC  R: AACCTCAGCCTCCAGAGTTC |

* F: forward strand; R: reverse strand
